# Viremia and antibody dynamics following atypical porcine pestivirus infection: a cohort study of pigs with congenital tremor

**DOI:** 10.1186/s40813-026-00524-2

**Published:** 2026-05-28

**Authors:** Frida Håjen Aae, Mette Myrmel, Birgit Ranheim, Mamata Khatri, Anna Bergfeldt, Maria Stokstad

**Affiliations:** 1 Department of Production Animal Clinical Sciences, Faculty of Veterinary Medicine, Norwegian University of Life Sciences, Postboks 5003, Ås, 1432 Norway; 2 Virology Unit, Faculty of Veterinary Medicine, Norwegian University of Life Sciences, Postboks 5003, Ås, 1432 Norway; 3 Norwegian Meat and Poultry Research Centre, Animalia AS, Postboks 396, Økern, Oslo, 0513 Norway; 4 Department of Preclinical Sciences and Pathology, Faculty of Veterinary Medicine, Norwegian University of Life Sciences, Postboks 5003, Ås, 1432 Norway

**Keywords:** Atypical porcine pestivirus, Cohort study, Congenital tremor, Humoral immunity, Viremia

## Abstract

Atypical porcine pestivirus (APPV) induces congenital tremor (CT) in piglets. Limited understanding of the relationship between viremia, antibody responses and clinical signs has impeded the development of effective preventive measures, as it is unclear which individuals are infectious and for how long. To characterize these aspects, a cohort field study with 43 piglets from herds experiencing CT and 21 external control piglets was conducted. CT-pigs and their healthy littermates were viremic at birth, with APPV RNA detected in serum by RT-qPCR, and viremia persisted in eight of the 15 pigs from CT-litters that remained in the study until four months of age, although levels declined over time. Pigs from unaffected litters in outbreak herds became viremic after litters were mixed. All pigs had APPV-specific anti-E2 antibodies, measured by an in-house ELISA, at three days of age. Levels declined by 59 days but were elevated again at four months of age in outbreak herd pigs. Lower viral loads were associated with higher antibody levels at this age, indicating an effective immune response. Although the duration of viremia was positively associated with the duration of tremors, 22% of pigs remained viremic after clinical signs had resolved. Furthermore, neither the amount of virus in serum nor antibody levels correlated with the degree of tremors. These findings reveal that piglets born with APPV-induced CT can remain viremic until four months of age and are able to produce APPV-specific antibodies. The absence or presence of tremors cannot be used to identify which pigs are viremic and thus at risk of transmitting the virus, highlighting a challenge for disease control.

**Supplementary Information:**

The online version contains supplementary material available at 10.1186/s40813-026-00524-2.

## Introduction

Atypical porcine pestivirus (APPV)-induced congenital tremor (CT) type A II is a neonatal disease reported from multiple countries, posing significant concerns for piglet welfare and productivity. Affected piglets display intention tremor, which can impair suckling and mobility, ultimately resulting in reduced growth and compromised overall health. The tremors place significant stress on the pigs, as they restrict voluntary movement, and can leave the animals immobilized while shaking intensely [1]. Although clinical signs usually diminish gradually after the first few days and weeks, tremors have been shown to persist until slaughter age or to reappear under stress [2–4]. The infection can occur during gestation, as APPV crosses the placenta to infect the foetuses [5]. In outbreaks, litters from gilts are often affected, indicating that protective immunity develops after initial exposure. In outbreaks, up to 85% of gilts and 20% of sows have been reported to farrow piglets with CT [5, 6]. This can have severe consequences, with mortality rates reported to reach 30% in affected litters [7].

The causative agent, APPV, was discovered about ten years ago [8], and has since been detected in pig industry worldwide [9–11]. CT and APPV-infections are likely under-reported due to the absence of routine surveillance programs and diagnostic tools specifically targeting APPV. Detection and surveillance are further limited since the discovery of APPV is recent and diagnostic tools, such as commercially available antibody tests, are not available. APPV is classified as species Pestivirus scrofae, belonging to the family Flaviviridae [ICTV, 12]. It is an enveloped, single-stranded, positive-sense RNA virus encoding one polyprotein that is processed into twelve mature proteins [8]. Among these, the E2 envelope glycoprotein is proposed to be the immune-dominant antigen [2] and enzyme-linked immunosorbent assays (ELISAs) targeting anti-E^rns^, -E2 and -NS3 antibodies have in the recent years been developed [2, 13, 14].

Other important and well-known livestock pestiviruses include bovine viral diarrhoea virus (BVDV), border disease virus (BDV) and classical swine fever virus (CSFV). Acute infection with these viruses during early gestation before developed immune competence can lead to persistently infected (PI) offspring with an absent or low antibody response and lifelong viral shedding. These are a major source of virus transmission and have important implications for herd management, biosecurity and vaccination strategies [15]. Determining whether APPV can also produce PI-animals is therefore an important step toward developing effective control measures. However, achieving this requires first addressing several knowledge gaps, including duration and dynamics of viremia, the humoral immune response, and their interplay in causing clinical outcomes of *in utero* or postnatal infections.

Most existing studies on APPV are cross-sectional with limited knowledge on how infection and immunity develop over time. Although a few studies on CT-piglets have reported prolonged viremia, findings on humoral immunity are inconsistent [2, 5, 7, 16]. It is unclear whether the prolonged viremia indicates APPV-tolerance, chronic infection despite a humoral immune response, latent infection with virus hiding in tissues, or reinfection. In addition, studies on infection and immunity dynamics lack detailed clinical evaluations. It is unknown whether some APPV-infected pigs present with mild or no tremors while shedding large amounts of virus, or conversely, display severe tremors with limited viral shedding.

To address these uncertainties, longitudinal studies on infected pigs are essential to advance knowledge of the relationship between clinical signs and infection dynamics. Ultimately, this could help guide disease control efforts and thereby improve animal welfare and production. The objectives of the present study were to (1) characterize viremia in pigs from herds with APPV-induced CT outbreaks, (2) assess the humoral immune response in these pigs, and (3) compare virological and serological findings with clinical outcomes.

## Materials and methods

### Study design

The study was conducted as a field study of naturally occurring congenital tremor. Cohorts of piglets from four herds with acute outbreak and one healthy control herd were followed from birth to approximately four months of age. The inclusion criterium for the outbreak herds was tremors in more than two litters in the farrowing group. For the control herd the inclusion criteria were no tremor observed during the last ten years and serum samples from 5 to 10 sows being APPV-negative on RT-qPCR. No assay for APPV-antibodies was available for the pre-study sampling. The present work was embedded in a broader CT-project (PestiPig) and characterization of clinical signs have been published by Aae et al., 2024 [17].

**Fig. 1:**
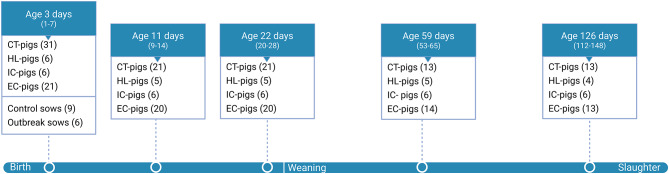
Timeline with number of pigs (n) and age at sampling time points. At each time point, pigs underwent clinical examination and serum sampling for APPV RT-qPCR and antibody ELISA. Congenital tremor (CT)-pigs: pigs with tremors at the first visit; Healthy littermates (HL)-pigs: pigs without tremors in litters with CT; Internal control (IC)-pigs: pigs from healthy litters in herds with CT-outbreak; and external controls (EC)-pigs: pigs from a healthy control herd, tested by RT-qPCR at the initial time point

An overview of included pigs, with their herd and litter origins, is provided in A1 (Additional files). Individual pigs were selected to obtain a range of severity of CT. Piglets in affected litters were classified as CT-pigs with severe or mild tremor (*n* = 31) or healthy littermates (*n* = 6), piglets from unaffected litters in outbreak herds were classified as internal controls (*n* = 6) and piglets from the CT-free herd as external controls (*n* = 21). Individual pigs were examined at five time points: as soon as possible following birth, twice during the suckling period, day 59 and 126. After the final examination during the suckling period, pigs were commingled. Some pigs were lost due to death or euthanasia for a pathology study within the Pestipig project (Fig. 1) [18].

A timeline, including age and number of animals in each group, can be found in Fig. 1. At each time point, serum samples were obtained from all individuals, and a clinical examination was conducted, including weight and body condition scoring. Tremor assessment was conducted visually by the same observer each time after the pigs were placed on the floor outside the pen. Severity was classified into three levels: no tremor, mild tremor (small muscle contractions not interfering with voluntary movements) or severe tremor (pronounced muscle contractions interfering with voluntary movements). Between visits, the pigs were monitored by the farmer, and any signs of disease or treatment were recorded. Additionally, serum samples from nine and six sows in the control and outbreak herd(s), respectively, were collected at the initial visit (herd 1: *n* = 3, herd 2: *n* = 1, and herd 4: *n* = 2). Of these, four sows from the outbreak herds farrowed litters with CT, while two produced internal control litters. The sows were primiparous, except for one sow that was in her second parity.

Serum samples from all pigs were tested for anti APPV antibodies by ELISA at each sampling point. All serum samples from pigs in outbreak herds and from sows were screened by an APPV-specific RT-qPCR, whereas only the initial samples from external control pigs were tested. Herd and animal information, clinical examination and outcomes can be found in the published article [17].

### Serum sampling

For repeated collection, blood was drawn from the jugular vein, while for the 15 sows a single sample was taken from the subcutaneous abdominal vein [19]. Samples were immediately chilled, transported to the laboratory, centrifuged at 1700 x G for 10 min and the serum stored at -80 °C.

### RNA extraction and APPV RT-qPCR

RNA was extracted from serum using the QIAsymphony (Qiagen, Hilden, Germany) and the Virus/Pathogen Mini Kit with the Cellfree200_V7_DPS protocol. Due to varying sample volumes, 100–300 µl of each sample was used and diluted with phosphate-buffered saline (PBS) to a final volume of 300 µl. The elution volume was set to 60 µl and extracted RNA was stored at -80 °C until analysis.

RT-qPCR for the APPV NS5B coding region was conducted using the AriaMx Realtime PCR system (Agilent Technologies) with the RNA UltrasenseTM One-Step Quantitative RT-PCR system (Invitrogen, Thermo Fischer, MD, USA). Samples were run in parallel, with the same set of negative and positive controls in each run. The primers used were APPV-NS5B-303 F (5`-GTAGGGCGGATACAGAAATA-3`), APPV-NS5B-385R (5`-GGYACTTCCTCCATCATGG-3`), and the probe APPV-NS5B-336: (FAM-AAATATTGGAAATYYATTGACAATTTGAC-BHQ1) [20]. The final concentration was 400 nM for each primer and 200 nM for the probe. Two µl of RNA was used in a total volume of 20 µl. The thermal cycling conditions were 50 °C for 30 min, 95 °C for 2 min, and 40 cycles of 95 °C for 15s and 56 °C for 40s. RT-qPCR data were analysed using the Agilent AriaMx software (Agilent Technologies).

Assay efficiency was determined using a ten-fold dilution series of APPV RNA, generating a standard curve with an amplification efficiency of 104% (E = 1.04, R^2^ = 0.98, slope = -3.23). Using this standard curve, Cq values were converted to relative viral load according to the following formula:

$$\:\mathrm{N}1=\mathrm{N}2{(1+\mathrm{E})}^{{\mathrm{C}\mathrm{q}}_{2}{-\mathrm{C}\mathrm{q}}_{1}}$$

N1 represents the sample, N2 (positive control) was set to 1 for relative quantification, E was 1.04, Cq_2_ represented the Cq of the positive control, and Cq_1_ the Cq of the sample. All relative values were adjusted for the serum volume used for RNA extraction to account for differences in input material.

### Anti-APPV antibody ELISA

An in-house anti APPV-specific enzyme-linked immunosorbent assay (ELISA) was established for detection of anti E2 antibodies in serum samples. Clear Flat-Bottom Immuno Nonsterile MaxiSorp 96-well ELISA plates (Thermo Fisher Scientific, Cat. No. 442404) were coated with 100 µl of E2 antigen (recombinant protein expressed in CHO cells, 0.39 mg/ml, GenScript, Piscataway, NJ, USA), diluted in coating buffer to 0.3 µg/100 ul. The coating buffer was made with 1.5 g sodium carbonate and 2.93 g sodium bicarbonate dissolved in 1 L of distilled water, with a pH of 9.6. Plates were incubated overnight at 4 °C, the wells emptied and washed 3x using a buffer consisting of 1x PBS and 0.05% Tween-20. Blocking buffer [1x PBS and 1% bovine serum albumin (BSA)], was added, the plates were incubated at 37 °C for 1 h and then washed 4x with wash buffer. Serum samples were diluted 1:100 in the blocking buffer, and 100 µl was added to the wells. Samples were run in parallel, and one set of known control samples were included in all plates to verify assay performance. The plates were incubated for 1 h at 37 °C, followed by washing 3x with wash buffer. Rabbit Anti-Pig IgG (whole molecule)-Peroxidase antibody (Sigma-Aldrich, Cat. No. A5670) was diluted 1:40 000 in blocking buffer and 100 µl added per well. The plates were incubated for 1 h at 37 °C and washed 3x with wash buffer, before 100 µl 1-Step Ultra TMB ELISA substrate solution (Thermo Fisher Scientific, Cat. No. 34028) was added. Plates were incubated at room temperature in the dark for 30 min, and 50 µl Stop Solution (Thermo Fisher Scientific, Cat. No. N600) was added. Absorbance was measured immediately at 450 nm using the Spark Multimode Microplate Reader (Tecan, Switzerland). The reference wavelength was set to 630 nm. Sera were analysed in duplicates and mean values were used.

### Change point analysis

Traditionally, establishing reliable positive and negative controls have been essential for determining accurate ELISA cut-off values. However, for many emerging pathogens, such as APPV, identifying true negative samples is a challenge. To overcome this, changepoint analysis can be applied to determine the cut-off for positive samples [21]. The analysis was carried out in R (version 4.3.3) using the changepoint package. This package applies a mean-based segmentation model to detect statistically significant shifts in the absorbance levels. Prior to analysis, the absorbance values from all serum samples were arranged in ascending order. The function cpt.mean() was used to perform change-point detection. The Pruned Exact Linear Time (PELT) algorithm was selected, and the default likelihood-based test statistic was used in combination with the minimum Bayesian information criterion (MBIC) as the penalty method. Based on the changepoints detected, shifts in absorbance levels were identified. The lowest stable region prior to the first shift was interpreted as the background level, and levels below this were considered negative. Using the results from the change-point analysis, cut off for absorbance levels was set to 0.51.

### Statistical analysis

Statistical analyses were conducted using STATA/SE 17.0 (StataCorp, College Station, TX, USA), and results were considered significant at *p* < 0.05. Relative viral load values were log10-transformed prior to analysis, and the transformed variable (Log10RVL) was used in all models. In all the following mixed-effects models, herd and individual were included as random effects. Litter was evaluated as an additional random effect but explained negligible variance and was excluded from final models.

A mixed-effects linear regression model was used to analyse differences in Log10RVL between clinical groups. Clinical group was included as fixed effect. Pairwise comparisons of adjusted means were performed and an interaction model with age as fixed effect was evaluated to assess time-dependent differences.

Absorbance values at the initial sampling were compared between herds using two-sample t-tests, with Bonferroni correction applied for multiple pairwise comparisons. Longitudinal changes in absorbance were analysed using mixed-effects linear regression with clinical group and age as fixed effects. Adjusted means and pairwise comparisons were derived from the fitted models.

The association between viral load and antibody levels was examined using two mixed-effects models. In the first model, absorbance was specified as the outcome, with Log10RVL, age and their interaction included as fixed effects. In the second model, Log10RVL was specified as the outcome, with absorbance, age and their interaction included as fixed effects. Age-specific associations were derived from the fitted models.

Linear regression was used to assess the associations between absorbance levels at the final visit and duration of viremia, and between age at last observed tremor and age at last detected viremia.

Mixed-effects linear regression models were used to examine associations between Log10RVL or absorbance, and the outcomes weight and body condition score (BCS). Log10RVL or absorbance and age were included as fixed effects. Age-specific associations were derived from the fitted models.

## Results

### Detection of APPV genomes

**Fig. 2:**
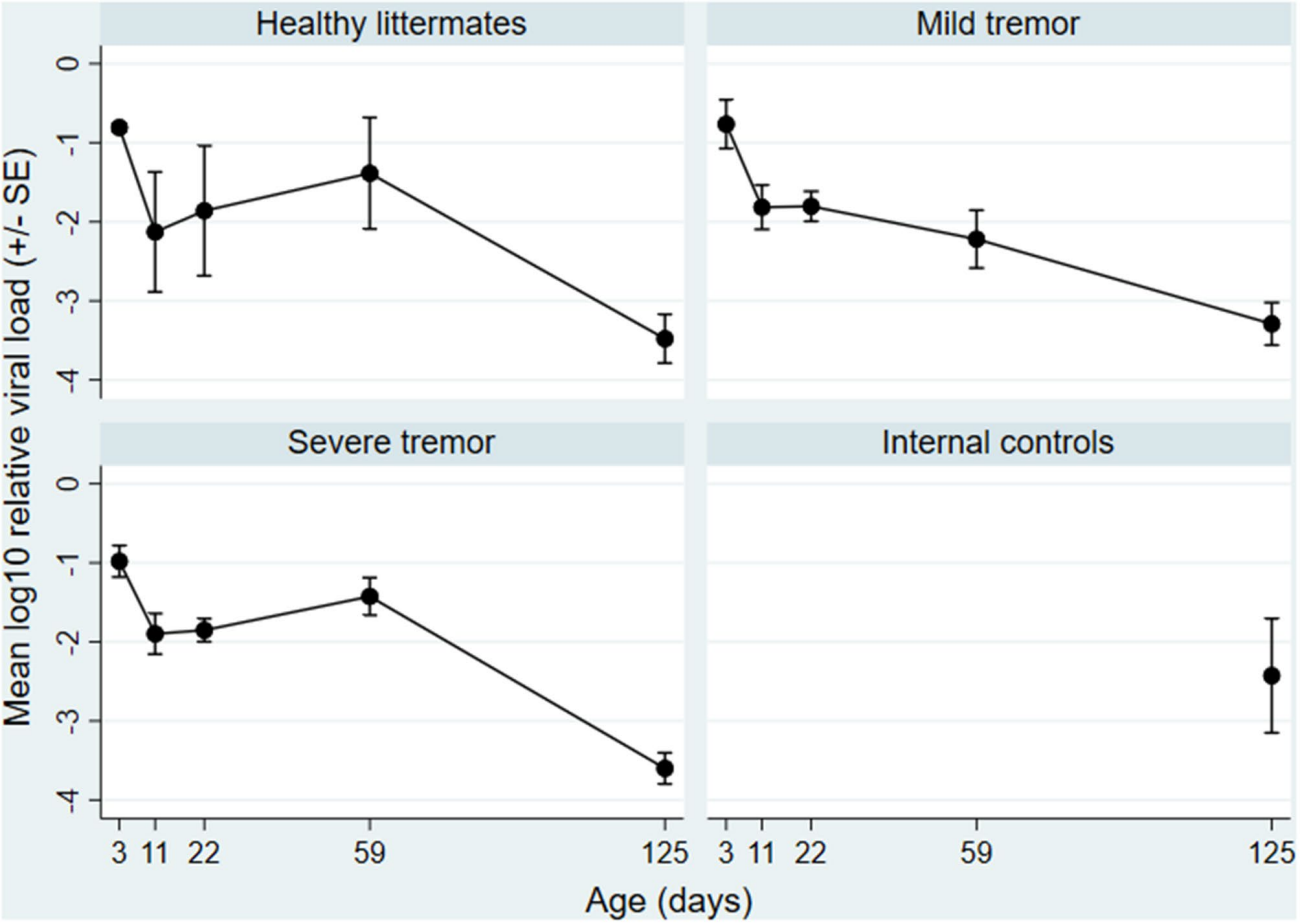
Mean log10 relative viral load (RVL) values in serum from pigs in outbreak herds at different attained ages (days). The Y-axis shows log10-transformed viral loads expressed relative to a positive control, where 0 indicates a viral load equal to the positive control and increasingly negative values indicate proportionally lower viral loads. Only pigs with detectable APPV levels were included in the graph. Error bars represent the standard error of the mean. Sample sizes across the five time points were: Healthy littermates (*n* = 3, 3, 3, 2, 2); Mild tremor (*n* = 7, 5, 5, 5, 3); Severe tremor (*n* = 24, 16, 16, 8, 3); and Internal control (*n* = 0, 0, 0, 0, 4). Healthy littermates: healthy pigs in CT-litters; Mild tremor: pigs with mild tremor in CT-litters; Severe tremor: pigs with severe tremor in CT-litters; Internal control: healthy pigs from healthy litters

**Fig. 3:**
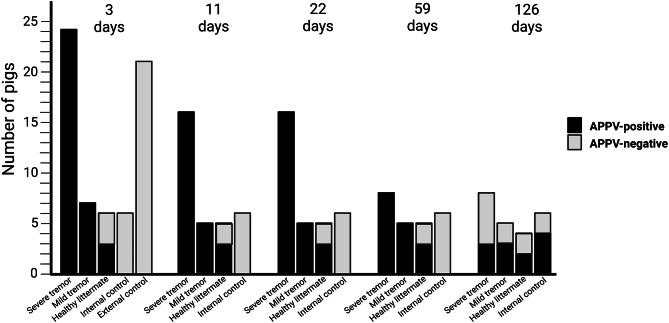
Number of APPV positive and negative pigs in each clinical group at each attained age. Severe tremor: pigs with severe tremor in congenital tremor (CT)-litters; Mild tremor: pigs with mild tremor in CT-litters; Healthy littermates: healthy pigs in CT-litters; Internal control: healthy pigs from healthy litters; External control: pigs from a healthy control herd

The development of viral load over time is shown in Fig. 2. Pigs from CT-litters had high viral RNA levels in the early samples, with declining levels over time. Three of the six healthy littermates were virus-positive in the initial samples, and two additional became positive during the suckling period. None of the internal control pigs tested positive during the suckling and weaner periods, but four out of six became virus-positive by four months of age (Fig. 3). All external control pigs and all sows tested negative for the APPV-genome.

A mixed-effects linear regression model showed no significant difference in relative viral load between CT-pigs with severe tremor, CT-pigs with mild tremor, and healthy littermates at any time point (*p* = 0.343).

Viremia persisted until four months of age in 53% of pigs (*n* = 8/15). The oldest pig testing positive was 136 days of age; however, no subsequent testing was performed beyond this time point (Table 1).

**Table 1:** Duration of viremia

Clinical group	Age at first APPV positive sample (days)		Age at last APPV positive sample (days)
Severe tremor CT-pig	2	112	
Severe tremor CT-pig	4	59	
Severe tremor CT-pig	2	57	
Severe tremor CT-pig	2	136	
Severe tremor CT-pig	3	64	
Severe tremor CT-pig	4	65	
Severe tremor CT-pig	1	63	
Severe tremor CT-pig	1	114	
Mild tremor CT-pig	4	114	
Mild tremor CT-pig	2	136	
Mild tremor CT-pig	4	65	
Mild tremor CT-pig	1	114	
Mild tremor CT-pig	1	63	
Healthy littermate	2	112	
Healthy littermate	4	114	
Healthy littermate	65	65	
Internal control	115	115	
Internal control	117	117	
Internal control	137	137	
Internal control	114	114	

Age at first and last positive sample in pigs grouped by clinical status. Only pigs from outbreak herds that were sampled until four months of age are included (*n* = 20)

### Anti APPV Antibodies

**Fig. 4:**
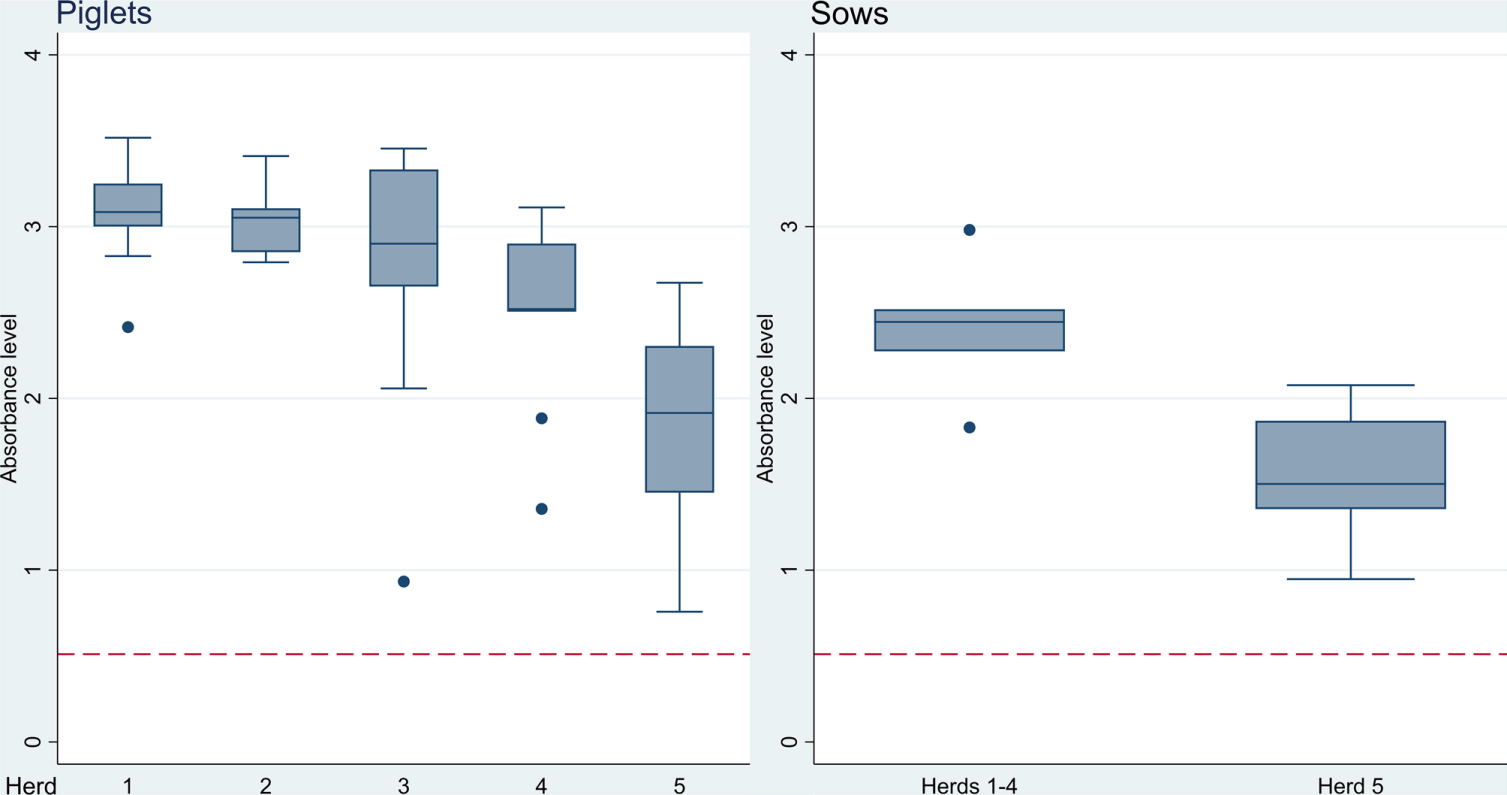
Serum anti APPV antibody ELISA absorbance levels. Left box plot: Absorbance levels in serum samples from 3 d old (range: 1–7 d) piglets from the outbreak herds (1 (*n* = 10, 2 (*n* = 9), 3 (*n* = 13) and 4 (*n* = 11)) and the control herd (5 (*n* = 21)). Right box plot: Absorbance levels in serum samples from sows from outbreak herds (herd 1–4 (*n* = 6)) compared to sows from the control herd (herd 5 (*n* = 7)). Each box represents the 25th to the 75th percentile of values in each group, and the horizontal line within the box shows the median value. The whiskers include values within 1.5 times the percentile range, and outliers are marked as dots

All piglets (1–7 days old) were APPV antibody positive in serum. Among piglets from outbreak herds, ELISA absorbance levels ranged from 0.93 to 3.52, whereas external control piglets had levels ranging from 0.76 to 2.67 (Fig. 4).

At the first visit, piglets from outbreak herds had significantly higher absorbance levels compared to external control piglets (mean = 2.83 vs. 1.83; t(62) = 6.87, *p* < 0.001). Pairwise comparisons confirmed higher absorbance levels in each outbreak herd compared to the control herd (all *p* < 0.01). Within outbreak herds, there were no statistically significant differences between herd 1 (mean = 3.07) and 2 (mean = 3.03), herd 1 and 3 (mean = 2.79), herd 2 and 3, or herd 3 and 4 (mean = 2.51) (all *p* > 0.26). Herd 1 and 2 had significantly higher absorbance levels than herd 4 (t(19) = 3.11, *p* = 0.006 and t(18) = 2.96, *p* = 0.009, respectively).

All sows were APPV antibody positive. Sows from outbreak herds showed significantly higher absorbance levels compared to the control herd (mean = 2.42 and 1.53, respectively, t(11)=-4.30, *p* = 0.0013; Fig. 4).

**Fig. 5:**
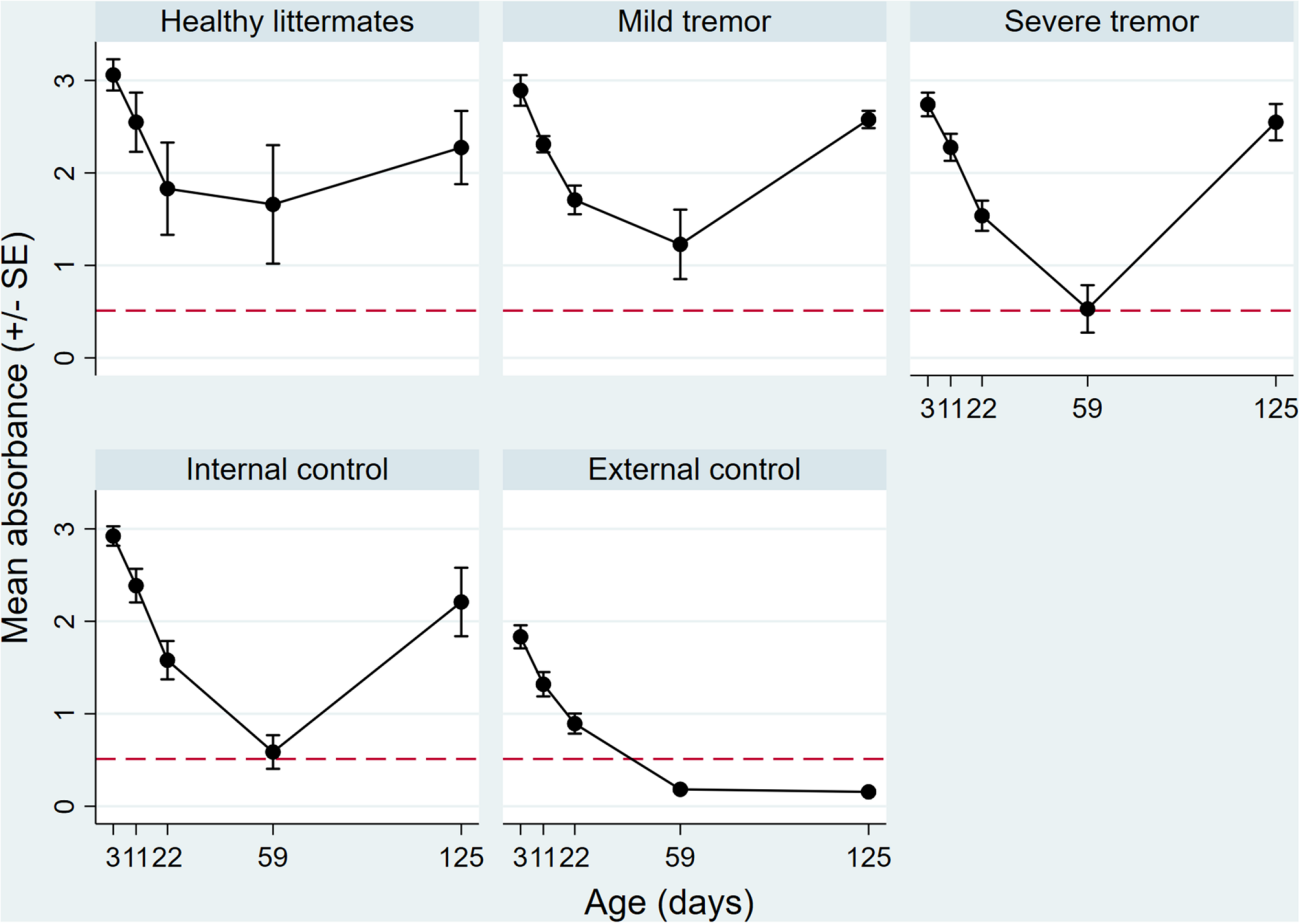
Mean serum anti APPV antibody ELISA absorbance levels over time with standard error bars. The cut off for positive samples is 0.51, represented with a horizontal dashed line. Healthy littermates: healthy pigs in CT-litters; Mild tremor: pigs with mild tremor in CT-litters; Severe tremor: pigs with severe tremor in CT-litters; Internal control: healthy pigs from healthy litters; External control: pigs from a healthy control herd. Sample sizes across the five time points were: Healthy littermates (*n* = 5, 4, 4, 4, 3); Mild tremor (*n* = 8, 6, 6, 6, 6); Severe tremor (*n* = 24, 16, 16, 8, 8); Internal control (*n* = 6, 6, 6, 6, 6); and external control (*n* = 21, 20, 20, 14, 12)

A mixed-effects linear regression model showed that the absorbance level did not differ between CT-pigs with severe tremor, CT-pigs with mild tremor, healthy littermates, and internal control pigs at any attained age (all *p* = 0.23–0.67). In contrast, external control pigs had significantly lower absorbance values than pigs from outbreak herds overall (*p* < 0.001). In this latter group, absorbance levels varied over time, with high levels at day 3, a decline by day 22–59, and a subsequent increase by day 126 (Fig. 5). Pairwise comparisons of model-adjusted means showed no significant differences between outbreak-herd pigs and external controls at 59 days (all *p* > 0.21), whereas all pigs from outbreak herds had higher absorbance values than external controls at 126 days (all *p* < 0.001). Mean absorbance and relative viral load in pigs by clinical group at each attained age can be found in Table 2.

**Table 2:** Mean serum absorbance levels and relative viral load in pigs grouped by tremor severity

Groups	Age in days	Mean absorbance value	Mean relative viral load
Severe tremor (*n* = 24)	3	2.7	+++ (*n* = 24)
Mild tremor (*n* = 7)	3	2.9	+++ (*n* = 7)
Healthy littermate (*n* = 6)	3	3.0	+++ (*n* = 3)
Internal controls (*n* = 6)	3	2.9	- (*n* = 6)
External controls (*n* = 21)	3	1.8	- (*n* = 21)
Severe tremor (*n* = 16)	11	2.3	++ (*n* = 16)
Mild tremor (*n* = 5)	11	2.4	++ (*n* = 5)
Healthy littermate (*n* = 5)	11	2.5	++ (*n* = 3)
Internal controls (*n* = 6)	11	2.4	- (*n* = 6)
External controls (*n* = 20)	11	1.3	
Severe tremor (*n* = 16)	22	1.5	++ (*n* = 16)
Mild tremor (*n* = 5)	22	1.7	++ (*n* = 5)
Healthy littermate (*n* = 5)	22	1.8	++ (*n* = 3)
Internal controls (*n* = 6)	22	1.6	- (*n* = 6)
External controls (*n* = 20)	22	0.9	
Severe tremor (*n* = 8)	59	0.5	++ (*n* = 8)
Mild tremor (*n* = 5)	59	1.2	++ (*n* = 5)
Healthy littermate (*n* = 5)	59	1.6	++ (*n* = 3)
Internal controls (*n* = 6)	59	0.6	- (*n* = 6)
External controls (*n* = 14)	59	0.2	
Severe tremor (*n* = 8)	126	2.6	+ (*n* = 3)
Mild tremor (*n* = 5)	126	2.5	+ (*n* = 3)
Healthy littermate (*n* = 4)	126	2.4	+ (*n* = 2)
Internal controls (*n* = 6)	126	2.2	+ (*n* = 4)
External controls (*n* = 13)	126	0.2	-

Relative viral load (RVL) was categorized as follows: - = negative,+ = Log10RVL ≤ -2.4, ++ = Log10RVL − 2.3 to -1.1, and +++ = Log10RVL > -1.1. Severe tremor: pigs with severe tremor in congenital tremor (CT)-litters; Mild tremor: pigs with mild tremor in CT-litters; Healthy littermates: healthy pigs in CT-litters; Internal control: healthy pigs from healthy litters; External control: pigs from a healthy control herd

### Relationship between viral load, antibody levels and clinical outcomes

**Fig. 6:**
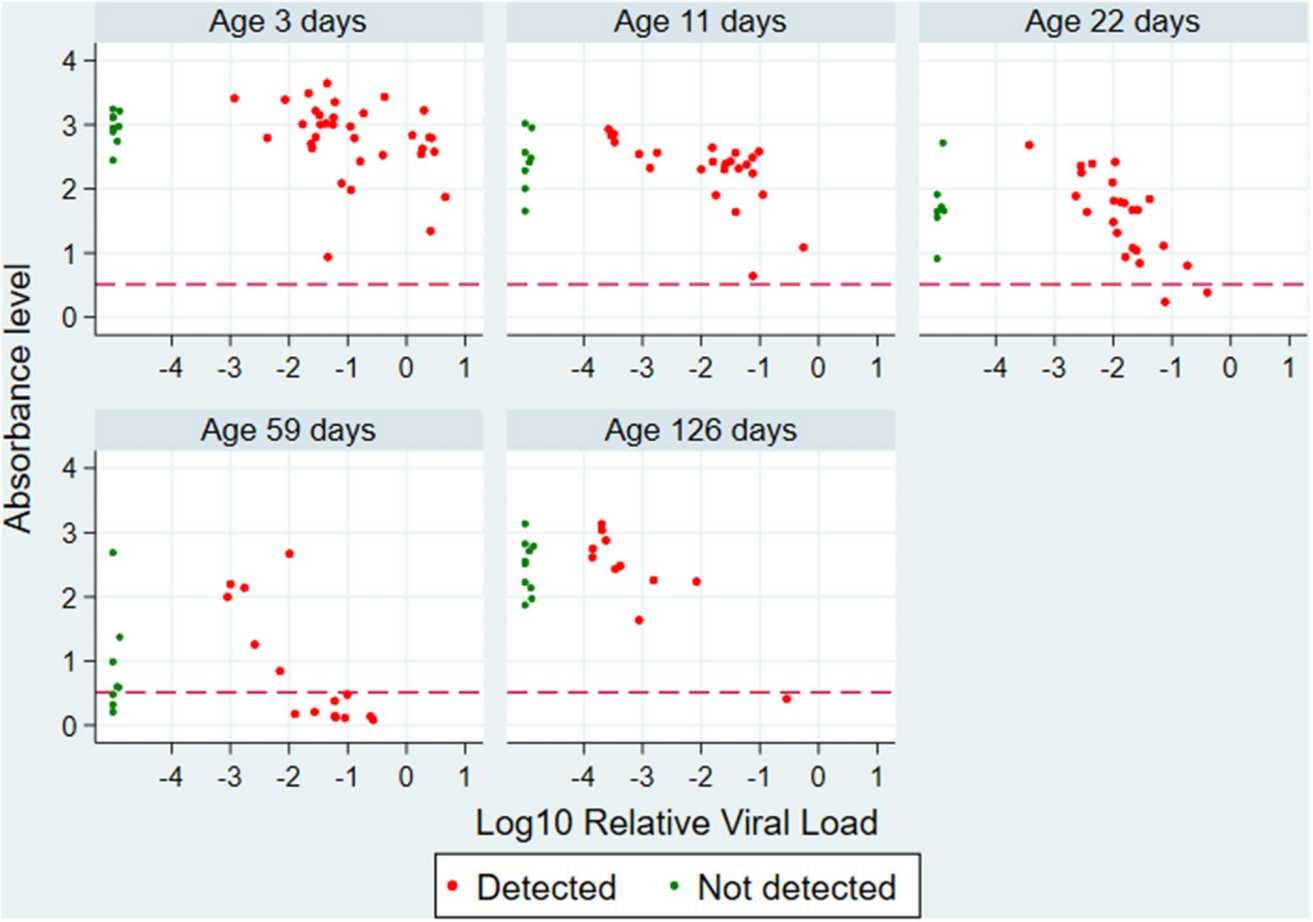
Scatterplot of antibody levels and Log10 relative viral load. The plot is showing the relationship between ELISA absorbance (antibody levels) and Log10 relative viral load (Log10RVL) from APPV RT-qPCR in serum samples from piglets in outbreak herds, as they grow older. The Log10RVL is calculated relative to a positive control, with Log10 = 0, and increasingly negative values indicate proportionally lower viral loads. Samples with no detectable virus are marked green. Positive samples are red. The horizontal dotted red line represents the absorbance cut off (0.51)

Higher antibody levels were generally associated with lower viral loads in pigs from outbreak herds, although there was some variability (Fig. 6). In the linear mixed-effects model with absorbance as the outcome, lower viral load was significantly associated with higher antibody levels at all examined ages. In the reversed analysis, where viral load was treated as the outcome, higher absorbance levels were also significantly associated with lower viral loads at all ages. Age-specific regression coefficients and p-values from both models are presented in Table 3.

**Table 3:** Regression coefficients and p-values for mixed model linear regression

Age (days)	Effect of Log10RVL on absorbance Regression coefficient	*p*-value	Effect of absorbance on Log10RVL Regression coefficient	*p*-value
3	-0.30	0.001	-0.55	0.002
11	-0.32	0.001	-1.23	< 0.001
22	-0.74	< 0.001	-0.80	< 0.001
59	-0.89	< 0.001	-0.68	< 0.001
126	-0.65	< 0.001	-1.22	< 0.001

Columns two and three show the estimated regression coefficients and p-value with Log10 relative viral load (Log10RVL) as the independent variable and absorbance level as the dependent variable. In columns four and five, absorbance level is treated as the independent variable and Log10RVL as the dependent variable. All estimates are derived from mixed-effects models including herd and individual as random effects

**Fig. 7:**
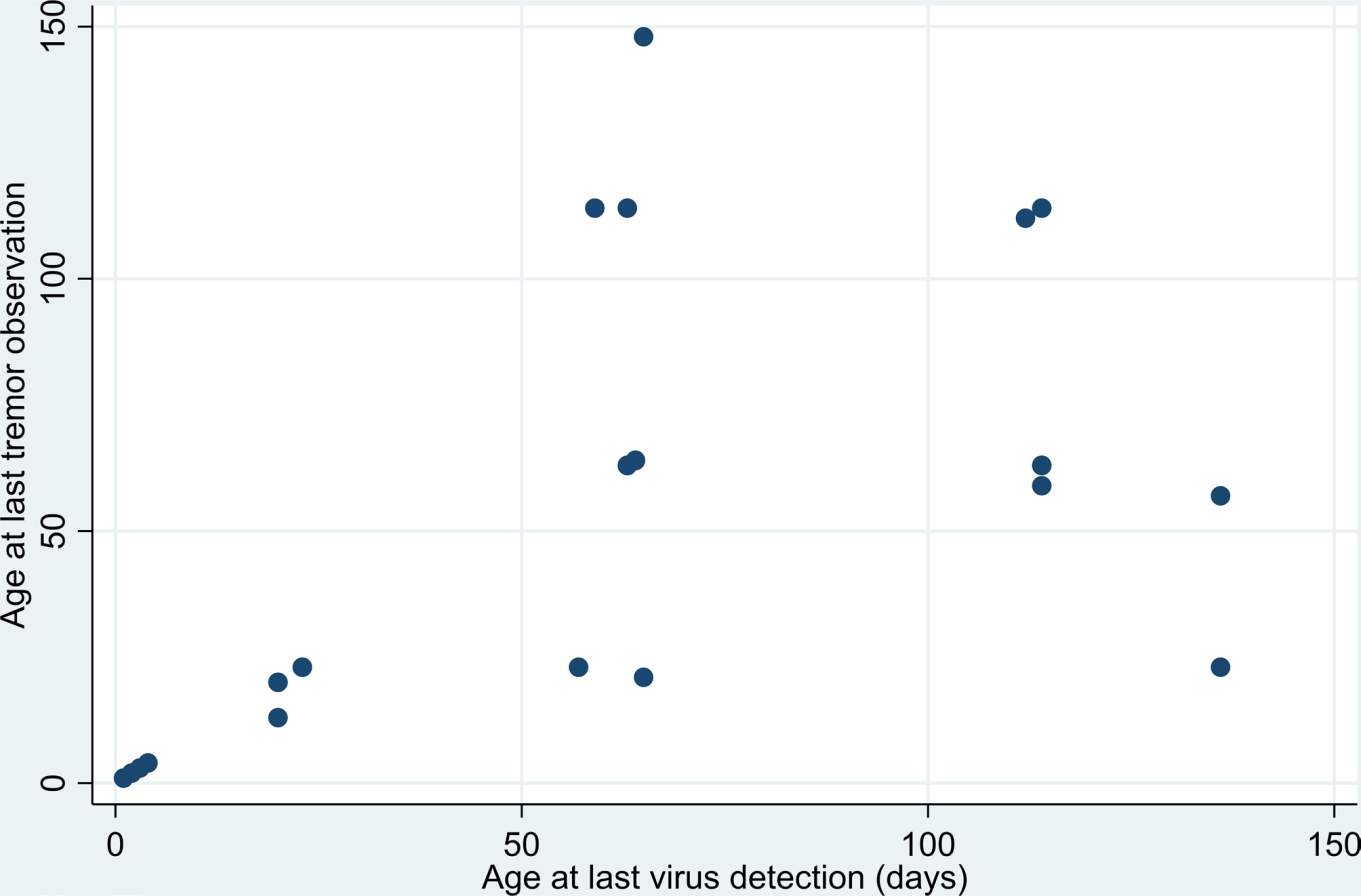
Scatterplot comparing oldest age with tremor and oldest age with viremia. The plot is showing the age at which tremors were last observed and the age at which virus was last detected in serum by RT-qPCR. Each point represents one piglet

Absorbance levels at the last visit did not predict the duration of viremia (b = 12.31, *p* = 0.51). The age at which tremors were last observed correlated positively with the age at which APPV RNA was last detected in serum (b = 0.66, *p* = 0.001, Fig. 7). However, there was considerable variability, with 9% (*n* = 3) piglets clearing the virus early while continuing to show clinical signs, and 22% (*n* = 7) remaining viremic without exhibiting tremors.

A mixed-effects model showed no association between relative viral load and weight at any visit (*p* = 0.23). There was a small but consistent negative association with body condition score (BCS) across visits (b=-0.12, *p* = 0.003). Antibody levels were not associated with weight (*p* = 0.08) but showed a positive association with BCS (b = 0.15, *p* = 0.001). No clinical signs of disease were observed in pigs that transitioned from virus-negative to virus-positive status during the study period, based on clinical assessments at each visit and the farmer’s health records. However, transient or mild signs occurring between visits cannot be excluded.

## Discussion

Pigs born in CT-litters had detectable APPV RNA in serum from the initial sampling point to four months of age. Viral load decreased over time, and all CT-pigs had detectable levels of antibodies at four months of age. The consistent detection of APPV in all piglets with CT at the first sampling strongly supports that *in utero* infection is a prerequisite for clinical disease to occur. Subclinical infections were also observed, with virus-positive healthy littermates exhibiting viral and antibody dynamics similar to CT-affected pigs. Transient viremia, typically caused by horizontal transmission [2], was not observed in these piglets, further supporting *in utero* infections. In contrast, some pigs that initially tested virus-negative later became positive, which is consistent with horizontal transmission after birth. Such postnatal infections were not accompanied by tremors or other observable health impairments, such as fever or reduced general condition. This supports that horizontal infection gives few or no clinical signs.

At the initial sampling, all piglets were APPV-antibody positive, with higher antibody levels in piglets from outbreak herds compared with those from the control herd. This reflects the presence of maternally derived antibodies acquired through colostrum, as piglets are born without circulating immunoglobulins, and corresponded closely to antibody levels measured in serum samples from the sows. All sows, including those from the healthy control herd, were antibody-positive but virus-negative, indicating prior exposure to APPV. Antibody levels were higher in sows from outbreak herds, suggesting a more recent infection compared with sows from the control herd. In the control herd, gilts were recruited from a single supplier. It is likely that APPV circulated within this source herd, and that the gilts experienced a transient viremia and subsequently seroconverted before arriving at the control herd. These observations also underline the challenge in identifying truly APPV-negative herds in studies of natural infections in the field and highlight the importance of robust approaches for interpreting serological data, such as change point analysis, when reliable negative controls are unavailable.

In outbreak herds, antibody levels were initially high, declined and subsequently increased, reflecting the transition from waning maternal antibodies to the piglet’s active immune response to an APPV infection [22–24]. In contrast, antibody levels in the control herd declined without a subsequent increase, consistent with the absence of APPV exposure. The increase in APPV-specific antibodies in CT-pigs at the final sampling indicates they were not PI-animals, as individuals immunotolerant to other pestiviruses fail to mount such antibody response [15]. Similar antibody patterns in young pigs have been reported in two previous studies on natural CT outbreaks. However, Buckley et al. [16] observed low antibody levels at slaughter age, while Cagatay et al. [2] reported more variable responses. Differences in antibody levels at slaughter age may reflect variation in infection pressure between herds, with higher levels of circulating virus promoting stronger antibody responses. Alternatively, differences in the timing of *in utero* infections, which is unknown for all three studies, could influence the outcomes, including differences in antibody production in adult animals. Although the ELISA cut-off used in this study was higher than in some previous reports [14, 25], a cut-off of 0.5 has been applied previously [2], and this conservative threshold did not limit our ability to identify antibody-positive animals, as serological responses were readily detected across cohorts and age groups.

The prolonged detection of APPV RNA together with a specific antibody response point to reinfection or possibly chronic infections, with the virus evading the immune system. Either way, pigs with lower viral loads had higher antibody levels at 59 and 126 days of age, consistent with an active immune response limiting viral replication. Although viral loads declined over time, the outcome beyond the study period remains unknown and future studies should extend follow-up beyond four months to determine the duration of viremia in infected pigs.

Clinical signs of CT were not predictive of whether pigs were viremic or not. As viremia is associated with viral shedding and possible transmission in APPV infections [5, 7], this has important epidemiological implications. Previous studies on APPV have demonstrated viral shedding in salvia and faeces from viremic pigs and suggested this as a key route of transmission [5, 7]. In the present study, healthy littermates were APPV-positive at four months of age and pigs with mild tremor tended to have longer duration of viremia than pigs with severe tremor, demonstrating that degree of tremors is not a reliable indicator for APPV infectious status.

Although the duration of viremia was associated with the duration of clinical disease, the relationship was not consistent. Some pigs became APPV RNA negative while tremors persisted, whereas others remained viremic in the absence of tremors. Similar observations have been reported previously, with APPV detected in tissues such as cerebellum and lymph nodes several months after clinical signs had resolved [26]. Based on this, clinical signs cannot be used to predict which animals are contagious or not during an outbreak. Furthermore, we found no evidence that viral load or antibody levels correlated with the severity of clinical signs. This supports the view that other factors, such as the timing of infection relative to neurodevelopment, determine clinical manifestations. In addition, pigs with higher viral load had slightly lower body condition scores, suggesting a potential impact on growth and production. However, this association was modest and should be interpreted with caution given the limited sample size.

The timing of *in utero* APPV-infection may influence both clinical outcomes, persistence of viremia and production of specific antibodies, as demonstrated for other pestiviruses that generate PI-animals. As this study was conducted under natural conditions, the gestational stage at which infection occurred could not be controlled. Consequently, while no APPV PI-animals were identified, their occurrence cannot be excluded, particularly in light of the variable antibody responses reported in previous studies [2, 16].

Some limitations should be considered when interpreting the findings. Group sizes within cohorts and the duration of follow-up was limited by practical considerations. In addition, some CT-pigs with severe tremor and external control pigs were removed during the study period for a pathological study within the Pestipig project, and a few individuals were culled due to disease (Fig. 1). Finally, as this was conducted as a field study, the infection status of herds prior to inclusion could not be fully controlled.

Despite these limitations, this study provides valuable insights by longitudinally following five distinct cohorts of pigs from five different herds, with parallel evaluation of viral load, antibody responses and clinical examinations. Although previous longitudinal field studies on APPV-infections have provided important insights, these studies have been conducted on a single farm, included only two or three cohorts, and typically classified clinical signs solely as the presence or absence of tremor. By contrast, the present study followed a larger number of pigs across five clinically defined cohorts, including CT-pigs with severe tremor, CT-pigs with mild tremor, healthy littermates, internal controls from unaffected litters within outbreak herds, and external controls from a CT-free herd. This broader cohort structure allowed for more detailed investigations of the relationship between infection dynamics and clinical presentations, while expanding the range of herd populations represented in the literature.

Further controlled experimental infection studies, including pre-colostral serum sampling and longitudinal monitoring of offsprings, could help clarify whether APPV PI-animals occur and to better understand their potential role in APPV epidemiology.

## Conclusion

This study provides a comprehensive characterization of APPV viremia, antibody response, and clinical outcomes in naturally infected pigs. Pigs born with CT and healthy littermates had prolonged viremia and specific antibody responses, indicating chronic infection or reinfection. The antibody responses likely limited viral replication. Although no persistently infected virus tolerant individuals were detected in this study, their existence in APPV-infections cannot be ruled out as timing of infection during gestation was unknown. The degree of tremor could not predict which individuals were viremic or for how long, and apparently healthy pigs carried the virus until four months of age. These findings strengthen the knowledge base for APPV surveillance and control and highlight the need for future experimental infections studies.

## Supplementary Information

Below is the link to the electronic supplementary material.

Supplementary Material 1: Additional file 1: Distribution of pigs between herds and litters. Table showing origin of piglets from the different litters and herds and number of pigs in the first and last sampling point

Supplementary Material 2: Additional file 2: Dataset. The following variables are included: Id: individual number for each pig; Litter: individual number for each litter from which pigs were enrolled; Herd: individual number for each herd included; Sampling point: 1 to 5; Age: age of each pig at current sampling point; CT_score_first: congenital tremor score at the initial sampling point (0 = healthy littermate, 1 = mild tremor CT-pig, 2 = severe tremor CT-pig, 3 = internal control pig, 4 = external control pig); CT_score: congenital tremor score at the current sampling point (0 = no tremor, 1 = mild tremor, 2 = severe tremor); Serumvolume: input volum of serum in RNA-extraction; Cq_raw: RT-qPCR Cq values from serum samples at the current sampling point; Cq_positive_control: RT-qPCR Cq values from the positive control; N1: calculation of relative viral load; Relative_viral_load: Relative viral load after adjusting for different input volume of serum; Log10RVL: Log10-transformed relative viral load; Absorbance: absorbance levels from APPV-specific anti-E2 ELISA at the current sampling point; BCS: body condition score at current sampling point; Weight: body weight (kg) at current sampling point; Arthritis: clinical signs of arthritis at current sampling point (0 = no, 1 = yes)

## Data Availability

All data generated or analysed during this study are included in the published article and its supplementary information files (A2).
