# Supplementary material for: Viremia and antibody dynamics following atypical porcine pestivirus infection: a cohort study of pigs with congenital tremor
Source: Porcine Health Manag. 2026 May 28;12:41. doi: 10.1186/s40813-026-00524-2 (PMC13421751; doi:10.1186/s40813-026-00524-2)
Supplement: Supplementary file 1 — Supplementary Material 1: Additional file 1: Distribution of pigs between herds and litters. Table showing origin of piglets from the different litters and herds and number of pigs in the first and last sampling point [file 40813_2026_524_MOESM1_ESM.docx]

A1: Distribution of pigs between herds and litters

| Herd | Litter | Type of litter | First visit | Last visit |
| --- | --- | --- | --- | --- |
| 1 | 1 | *Congenital tremor | 3 pigs | 2 pigs |
|  | 2 | Congenital tremor | 5 pigs | 3 pigs |
|  | 3 | Internal control | 1 pig | 1 pig |
|  | 4 | Internal control | 1 pig | 1 pig |
| 2 | 5 | Congenital tremor | 8 pigs | 3 pigs |
|  | 6 | Internal control | 1 pig | 1 pig |
| 3 | 7 | Congenital tremor | 4 pigs | 2 pigs |
|  | 8 | Congenital tremor | 3 pigs | 3 pigs |
|  | 9 | Congenital tremor | 4 pigs | 0 pigs |
|  | 10 | Internal control | 1 pig | 1 pig |
|  | 11 | Internal control | 1 pig | 1 pig |
| 4 | 12 | Congenital tremor | 10 pigs | 4 pigs |
|  | 13 | Internal control | 1 pig | 1 pig |
| 5 | 14 | External control | 7 pigs | 6 pigs |
|  | 15 | External control | 7 pigs | 1 pig |
|  | 16 | External control | 7 pigs | 6 pigs |
| Total number | |  | 64 pigs | 36 pigs |

*Congenital tremor litters comprised both CT-pigs and healthy littermates.
